# Development and psychometric properties of the “Suicidality: Treatment Occurring in Paediatrics (STOP) Risk and Resilience Factors Scales” in adolescents

**DOI:** 10.1007/s00787-019-01328-2

**Published:** 2019-05-03

**Authors:** A. Rodríguez-Quiroga, I. Flamarique, J. Castro-Fornieles, K. Lievesley, J. K. Buitelaar, D. Coghill, C. M. Díaz-Caneja, R. W. Dittmann, A. Gupta, P. J. Hoekstra, L. Kehrmann, C. Llorente, D. Purper-Ouakil, U. M. E. Schulze, A. Zuddas, R. Sala, J. Singh, F. Fiori, C. Arango, Paramala Santosh, The STOP Consortium, Alastair Sutcliffe, Sarah Curran, Laura Selema, Robert Flanagan, Ian Craig, Nathan Parnell, Keren Yeboah, Gideon Lack, Florence Pupier, Loes Vinkenvleugel, Jeffrey Glennon, Mireille Bakker, Cora Drent, Elly Bloem, Mark-Peter Steenhuis, Ruth Berg, Alexander Häge, Mahmud Ben Dau, Konstantin Mechler, Sylke Rauscher, Sonja Aslan, Simon Schlanser, Ferdinand Keller, Alexander Schneider, Paul Plener, Jörg M. Fegert, Jacqui Paton, Macey Murray, Noha Iessa, Alfred Kolozsvari, Helen Furse, Nick Penkov, Claire Baillon, Hugo Peyre, David Cohen, Olivier Bonnot, Julie Brunelle, Nathalie Franc, Pierre Raysse, Véronique Humbertclaude, Ana Espliego, Jessica Merchán, Cecilia Tapia, Immaculada Baeza, Soledad Romero, Amalia La Fuente, Ana Ortiz, Manuela Pintor, Franca Ligas, Francesca Micol Cera, Roberta Frongia, Bruno Falissard, Ameli Schwalber, Juliane Dittrich, Andrea Wohner, Katrin Zimmermann, Andrea Schwalber, Katherine Aitchison

**Affiliations:** 1 Child and Adolescent Psychiatry Department, Instituto de Investigación Sanitaria Gregorio Marañón (IiSGM), School of Medicine, Hospital General Universitario Gregorio Marañón, CIBERSAM, Universidad Complutense, Madrid, Spain; 2 Child and Adolescent Psychiatry and Psychology Department, 2014SGR489, Institute Clinic of Neurosciences, Hospital Clinic of Barcelona, CIBERSAM, Barcelona, Spain; 3 Centro de Investigación Biomédica en Red de Salud Mental, CIBERSAM, Madrid, Spain; 4 Department of Psychiatry and Clinical Psychology, University of Barcelona, Barcelona, Spain; 5 Department of Child and Adolescent Psychiatry, Institute of Psychiatry, Psychology and Neurosciences, King’s College London, London, UK; 6 HealthTracker Ltd, Gillingham, Kent UK; 7 Department of Cognitive Neuroscience, Donders Institute for Brain, Cognition and Behaviour, Radboud University Medical Centre, Nijmegen, The Netherlands; 8 Karakter Child and Adolescent Psychiatry University Centre, Nijmegen, The Netherlands; 9 Department of Paediatrics and Psychiatry, School of Medicine, Dentistry and Health Sciences, University of Melbourne, Melbourne, Australia; 10 Murdoch Children’s Research Institute, Melbourne, Australia; 11 Division of Neuroscience, School of Medicine, University of Dundee, Dundee, UK; 12 Paediatric Psychopharmacology, Department of Child and Adolescent Psychiatry, Central Institute of Mental Health (CIMH), Medical Faculty Mannheim, University of Heidelberg, Mannheim, Germany; 13 Department of Paediatric Respiratory Medicine, Kings College Hospital NHS Foundation Trust, Denmark Hill, London, UK; 14 Department of Paediatric Respiratory Medicine, Kings College London, London, UK; 15 Department of Child and Adolescent Psychiatry, University Medical Center Groningen, University of Groningen, Groningen, The Netherlands; 16 Hôpital Saint Eloi, Médecine Psychologique de l’Enfant et de l’Adolescent, CHRU Montpellier, Montpellier, France; 17 Department of Child and Adolescent Psychiatry/Psychotherapy, University of Ulm, Ulm, Germany; 18 Child and Adolescent Neuropsychiatry Unit, Department of Biomedical Sciences, University of Cagliari, Cagliari, Italy; 19 “A. Cao” Paediatric Hospital, “G. Brotzu” Hospital Trust, Cagliari University Hospital, Cagliari, Italy; 20 Centre for Interventional Paediatric Psychopharmacology and Rare Diseases, South London and Maudsley NHS Foundation Trust, London, UK

**Keywords:** Children, Adolescents, Suicidality, Risk, Resilience, Psychosocial, Questionnaire development and validation

## Abstract

Suicidality in the child and adolescent population is a major public health concern. There is, however, a lack of developmentally sensitive valid and reliable instruments that can capture data on risk, and clinical and psychosocial mediators of suicidality in young people. In this study, we aimed to develop and assess the validity of instruments evaluating the psychosocial risk and protective factors for suicidal behaviours in the adolescent population. In Phase 1, based on a systematic literature review of suicidality, focus groups, and expert panel advice, the risk factors and protective factors (resilience factors) were identified and the adolescent, parent, and clinician versions of the STOP-Suicidality Risk Factors Scale (STOP-SRiFS) and the Resilience Factors Scale (STOP-SReFS) were developed. Phase 2 involved instrument validation and comprised of two samples (Sample 1 and 2). Sample 1 consisted of 87 adolescents, their parents/carers, and clinicians from the various participating centres, and Sample 2 consisted of three sub-samples: adolescents (*n* = 259) who completed STOP-SRiFS and/or the STOP-SReFS scales, parents (*n* = 213) who completed one or both of the scales, and the clinicians who completed the scales (*n* = 254). The STOP-SRiFS demonstrated a good construct validity—the Cronbach Alpha for the adolescent (*α* = 0.864), parent (*α* = 0.842), and clinician (*α* = 0.722) versions of the scale. Test–retest reliability, inter-rater reliability, and content validity were good for all three versions of the STOP-SRiFS. The sub-scales generated using Exploratory Factor Analysis (EFA) were the (1) anxiety and depression risk, (2) substance misuse risk, (3) interpersonal risk, (4) chronic risk, and (5) risk due to life events. For the STOP-SRiFS, statistically significant correlations were found between the Columbia-Suicide Severity Rating Scale (C-SSRS) total score and the adolescent, parent, and clinical versions of the STOP-SRiFS sub-scale scores. The STOP-SRiFS showed good psychometric properties. This study demonstrated a good construct validity for the STOP-SReFS—the Cronbach Alpha for the three versions were good (adolescent: *α* = 0.775; parent: *α* = 0.808; *α* = clinician: 0.808). EFA for the adolescent version of the STOP-SReFS, which consists of 9 resilience factors domains, generated two factors (1) interpersonal resilience and (2) cognitive resilience. The STOP-SReFS Cognitive Resilience sub-scale for the adolescent was negatively correlated (*r* = − 0.275) with the C-SSRS total score, showing that there was lower suicidality in those with greater Cognitive Resilience. The STOP-SReFS Interpersonal resilience sub-scale correlations were all negative, but none of them were significantly different to the C-SSRS total scores for either the adolescent, parent, or clinician versions of the scales. This is not surprising, because the items in this sub-scale capture a much larger time-scale, compared to the C-SSRS rating period. The STOP-SReFS showed good psychometric properties. The STOP-SRiFS and STOP-SReFS are instruments that can be used in future studies about suicidality in children and adolescents.

## Introduction

Suicide is one of the major causes of death worldwide, with figures suggesting that approximately 1 million people commit suicide each year [1]. Although completed suicide is rare before the age of 10, suicidal behaviour increases sharply during adolescence and is a leading cause of death among young people [2]. Several biological, social, and psychological risk factors for suicidality seem to be shared by children, adolescents, and adults. Suicide risk follows a multifactorial trajectory and is increased in many psychiatric disorders varying by diagnosis, gender, and age [3]. The previous evidence suggests, however, that some risk factors for suicide might be different in adolescents compared with adults [4]. In adolescents, it is frequent that negative life events precede suicidal behaviour, most commonly family conflicts [5–7], changes of residence [8], romantic breakup [9], conflict with peers, including bullying [10, 11], and/or academic failure [12]. These differences in adolescence indicate a pressing need for the development of instruments aimed at specifically assessing protective and risk factors in young people. Considering that many of the adolescents committing suicide have never received any mental health support [13] and that several interventions have shown efficacy in preventing suicidal behaviour [14–16], it is essential to develop mechanisms that enable identification of subjects at risk and promote early intervention. Since suicide is a sensitive topic, which can be associated with stigma, web-based health monitoring platforms could be especially useful tools, as they provide a space for privacy.

To date, there are few valid and reliable, and developmentally sensitive instruments for collecting comprehensive data on risk, clinical and psychosocial mediators of suicidality in paediatric populations available for use by clinicians [17, 18]. One of the most widely accepted screening instruments, the Columbia-Suicide Severity Rating Scale (C-SSRS), has been shown to identify accurately individuals at risk of suicide, both in adult and paediatric populations, and has been used as the gold standard for the assessment of suicidal ideation and behaviours in clinical trials [19]; however, its clinical utility has been questioned [20]. One reason for this is that the C-SSRS has been deemed not to be sensitive enough to be able to capture the full range of suicidal ideation or behaviour [20]. The development of the STOP (Suicidality: Treatment Occurring in Paediatrics) Risk and Resilience Factors Scales has the potential to overcome this limitation by addressing the full range of suicidal ideation or behaviour whether singly or in combination.

The STOP project (Suicidality: Treatment Occurring in Paediatrics http://cordis.europa.eu/project/rcn/97369_en.html) was predominantly dedicated to the development of a comprehensive web-based assessment of suicidality and its mediators in children and adolescents. The aim of this specific study, which was embedded within the overall project, was to develop and assess the validity of the multi-informant STOP-Risk Factors Scale (STOP-SRiFS) and the multi-informant STOP Resilience Factors Scale (STOP-SReFS) as instruments for the collection of comprehensive data on psychosocial risk and protective factors for suicidal behaviours in the adolescent population.

## Methods

**Fig. 1:**
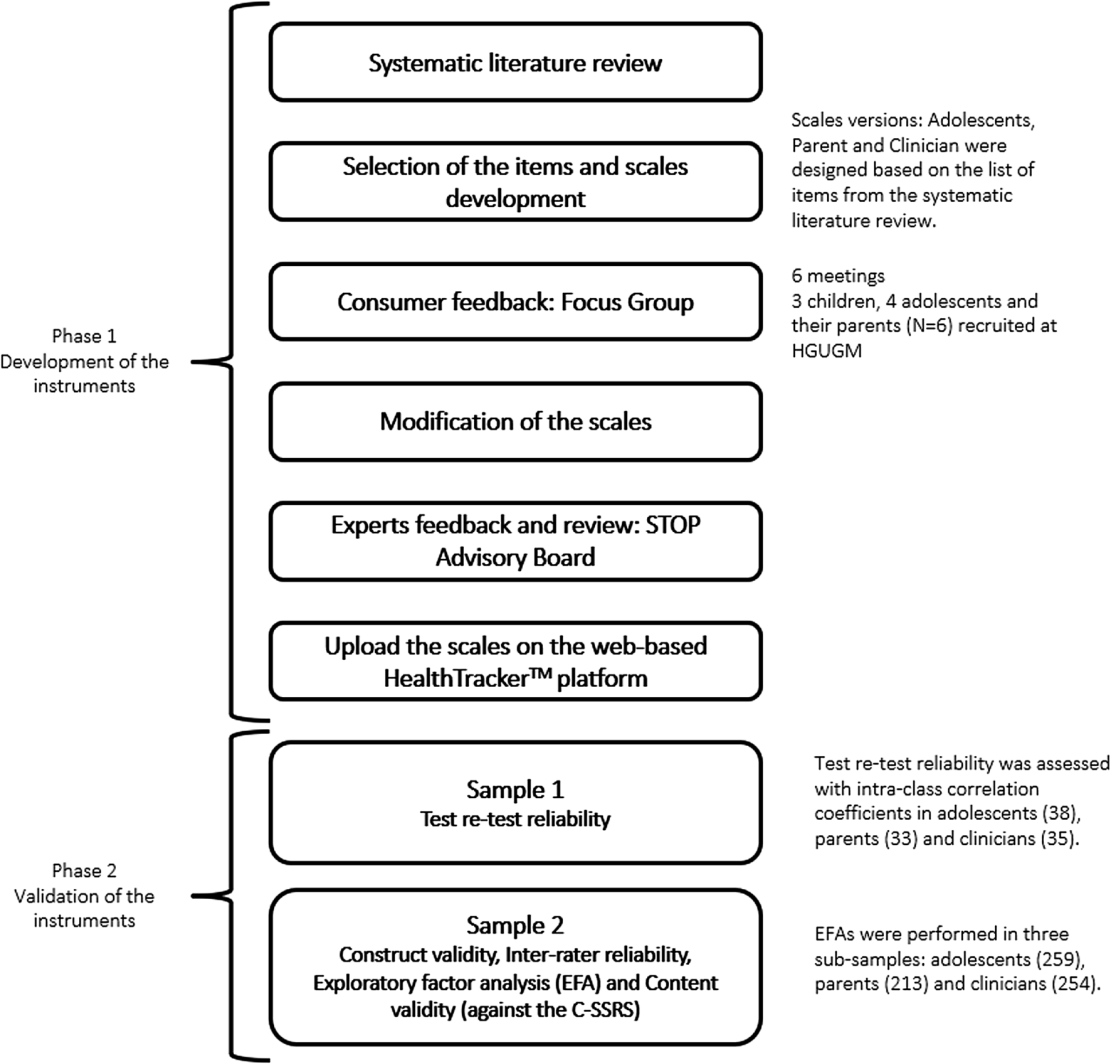
General overview of the development and validation of the STOP-Suicidality Risk Factors Scale (STOP-SRiFS) and the STOP-Suicidality Resilience Factors Scale (STOP-SReFS). *C-SSRS* Columbia-Suicide Severity Rating Scale, *EFA* exploratory factor analysis, *HGUGM* Hospital General Universitario Gregorio Marañón, Madrid, *STOP* Suicidality: Treatment Occurring in Paediatrics

Figure 1 shows a general overview of the development and validation of the STOP-SRiFS and the STOP-SReFS. Phase 1 focused on the development of the scales and Phase 2 focused on their validation. For Phase 2—sample 1 (*n* = 87), the scales were administered to a sample of adolescents, their parents/carers, and clinicians (Fig. 1). This sample served to explore the psychometric properties of the scales (test–retest reliability). Sample 2 consisted of adolescents (*n* = 259) that completed the STOP-SRiFS and/or the STOP-SReFS scales, parents (*n* = 213) who completed one or both scales, and the young persons’ clinicians (*n* = 254). The samples partially overlapped with one another. Sample 2 was used for the Exploratory Factor Analyses (EFA) and the other psychometric analyses of the scales.

Informed assent/consent was obtained from participants and/or their legal representatives, according to the ethical and legal standards in the participating countries. The study had approval from the Institutional Review Boards of all participating sites. Patients were recruited from the secondary/tertiary clinics from the various participatory Departments which were part of the project across the EU. The respiratory clinics from King’s College Hospital, London, and Evelina London Children’s Hospital contributed to the recruitment of the subjects with bronchial asthma and respiratory allergies. Those from the general population were identified through advertising on websites, schools, and libraries in the UK.

### Development of the scales

*Systematic literature review* A comprehensive and systematic literature review was performed at the outset of the study to identify the common and frequently reported risk and protective factors for suicidality in the paediatric population [21]. It also considered the aspects of suicidality that were covered by the C-SSRS [20] and other features relating to the revised nomenclature for the study of suicidal behaviours [22].

*Selection of items and Scale development* For both the STOP-SRiFS and STOP-SReFS, three versions (Adolescent, Parent and Clinician versions) were designed based on the list of domains extracted from the systematic literature review, input from focus groups, and expert feedback. The authors followed the U.S. Food and Drug Administration (FDA) recommendations for patient outcome measure development [23].

*Consumer feedback: focus groups* To explore patient’s views on risk and resilience factors of suicidality, identify new items, and to verify the understanding of the items, six meetings were carried out with children, adolescents, and parents (see Fig. 1). Each group session was conducted by two clinicians and was recorded with a video camera. Notes were taken during each focus group and reviewed by the experts. Based on the focus groups, some items were simplified, re-worded using age-appropriate vocabulary, or dropped; and answer options were reduced and converted to a 4-point scoring scale.

*Expert feedback: STOP scientific advisory board* The various experts in the study and the STOP scientific advisory board reviewed the draft versions of each scale and suggested minor modifications, which were incorporated into the final versions. The final versions of the scales in English and Spanish were reviewed by a professional translator. Following this, the English versions were then translated into German, Dutch, French, and Italian, and then back-translated into English. Clinicians from each participating country in the consortium ensured that the meaning of each statement remained culturally appropriate and meaningful.

*Upload of the scales to HealthTracker*^*TM*^ Once developed, the STOP-SRiFS and STOP-SReFS scales were uploaded onto the web-based HealthTracker™ platform, an e-health platform that includes a range of different scales for monitoring physical or emotional problems [21]. It was decided that the risk factors and protective factors would be presented sequentially as two scales, the STOP-Suicidality Risk Factors Scale (STOP-SRiFS) and the STOP-Suicidality Resilience Factors Scale (STOP-SReFS). At the end of this process, there are six scales; two for each different role that the scale can be assigned to/completed by. The scales are the STOP-ReFS for adolescent, parent, and clinicians, and the STOP-RiFS for adolescents, parents and clinicians.

### Scoring of the scales

The focus groups and the expert panel assisted in deciding the response options to the questions and also how to score the questions.

#### Scoring of the STOP-SRiFS

The majority of the items in the STOP-SRiFS were single questions, except for two items (“suicide on internet”, and “history of attempt”) that had two sub-questions each. The item on “suicide on Internet” dealt with (1) the number of times the adolescent had looked up information about suicidal behaviours or acts described in the item; and (2) when was the last time that they had searched the internet about this. The item on “history of attempt” dealt with (1) the number of times that they had attempted suicide in the past, and (2) when was the last attempt. For these two items, the score was obtained by the sum of the two scores divided by two. The score for each STOP-SRiFS questions ranged from 0 to 4. The undefined answers (“I don’t know”) were coded as 888 and then substituted with an empty cell.

#### Scoring of the STOP-SReFS

In the STOP-SReFS, each item was composed of two sub-questions: the first one dealt with the importance the adolescent gave to the item and the second one dealt with how useful the same item was in relation to protecting them against suicidality. The score of each item is obtained by the sum of the scores of the two sub-questions divided by two. The score for the STOP-SReFS items ranged from 0 (not at all) to 4 (a great deal). The undefined answers (“I don’t know”) were coded as 888 and then substituted with an empty cell. Each item score was given by the sum of the two questions which composed an item divided by two.

The scales scoring method allows the presence of undefined answers when a subject who filled the scales chose “I don’t know” as an answer. This was done to address the case in which the subject was unable to decide the answer, as forced answers are difficult when dealing with sensitive clinical issues such as suicidality. Little’s Missing at Random Tests for all the versions of the scales which were run before the estimation of the composite scores and for single question items. The results of those analyses showed that it was not possible to impute a value for the undefined answers, and therefore, those were left as empty (not given) answers.

### Phase 2: data analyses (validation of the instruments)

Subjects completed the questionnaires online using the web-based HealthTracker™ platform. SPSS version 23 [24] was used for the analyses.

*Sample 1* Consisted of 87 adolescents, their parents/carers, and clinicians from the various participating centres, who were re-administrated the scales within a maximum time of 3 weeks. This sample was used to test the time stability (test–retest reliability) of all versions of the STOP-SRiFS and STOP-SReFS.

*Sample 2* Consisted of 259 adolescents, 213 parents of adolescents, and 254 clinicians. Completion rates varied, because an adolescent might have completed the scale but not the parent of the adolescent or the clinician (see Table 2).

Construct validity using Cronbach’s alpha, test–retest reliability using correlations between repeat completions within 3 weeks, inter-rater reliability through correlations between the three versions of the scales, content and concurrent validity, through comparing the scores with that of the C-SSRS, and the sub-scales were generated using the Exploratory Factor Analysis (EFA) on the Adolescent, Parent, and Clinician versions of the scales. The sample sizes for all versions of both scales were above 200 and were considered adequate for these analyses [25]. The extraction method used was principal axis factoring, and Promax rotation was undertaken.

To assess the concurrent validity, the adolescent, parent, and clinician versions of the scales were correlated with the C-SSRS using Pearson’s correlations. The previous studies using the C-SSRS have shown convergent and divergent validity with other multi-informant suicidal ideation and behaviour scales and high sensitivity and specificity for suicidal behaviour [26].

## Results

Sample 1 comprised of 87 adolescents (mean age of 15.66 ± 1.66; 41.4% males and 58.6% females) (see Table 1 for the characteristics of Sample 1). Sample 2 was primarily composed of adolescents who had been screened as having some suicidality on the STOP 4-item Suicidality Screening questionnaire [21] and their parents and clinicians. The sample consisted of 259 adolescents (patient age at first assignment was 15.03 ± 1.599) who completed STOP-SRiFS and/or the STOP-SReFS scales; 213 parents (patient age at first assignment was 14.92 ± 1.797) who filled one or both of the scales; and 254 clinicians (patient age at first assignment was 15.17 ± 1.552) (see Table 2 for demographics of Sample 2).

**Table 1:** Description of Sample 1

*Gender*	
Male	36
Female	51
Total	87
*Ethnicity*	
Not Set	12
White	67
Asian	1
Chinese	2
Hispanic	5
Total	87
*Language*	
French (France)	20
German (Germany)	6
Italian (Italy)	28
Spanish (Spain)	33
Total	87

Developmental range was adolescent for all. Age 15.66 ± 1.66 years

**Table 2:** Description of Sample 2

	Adolescent sample	Parent sample	Clinician sample
*Treatment group*			
Aripiprazole	33	30	41
Cognitive behavioural therapy	41	41	64
Fluoxetine	68	61	86
General population	55	30	8
Montelukast	8	2	4
Other asthma or allergy med	10	3	1
Risperidone	44	46	50
Total	259	213	254
*Ethnicity*			
White	202	171	196
Asian	13	6	3
Black	7	6	8
Mixed	5	6	8
Arabic	1	1	–
Chinese	2	2	1
Hispanic	7	9	9
Gypsy	2	2	2
Ethnicity not set	20	10	27
Total	259	213	254
*Gender*			
Not set	–	1	1
Male	94	88	88
Female	165	124	165
Total	259	213	254

Version	Completions		
Adolescent	259		
Parent	213		
Clinician	254		

	Adolescent	Parent	Clinician
Number	259	213	254
Patient age at first assignment	15.03	14.92	15.17
Std. Deviation	1.599	1.797	1.552

### STOP-SRiFS

1. *Construct validity* The STOP-SRiFS Adolescent version demonstrated a good reliability (Cronbach’s *α* = 0.864) (Cronbach’s threshold was set at *α* > 0.700 [27]). For the Parent version of the scale, two items were excluded, because their Corrected Item-Total Correlation (CITC) was below the acceptance threshold [“Sexual Identity” (CITC = 0.056), and “Change of residence” (CITC = − 0.158)]. After excluding these two items, the STOP-SRiFS Parent version had good Cronbach’s alpha value (*α* = 0.842). Similarly, the STOP-SRiFS Clinician version showed a good Cronbach’s alpha (*α* = 0.722) when four items were excluded [chronic physical illness (CITC = − 0.066), being bullied (CITC = − 0.072), use of drugs (CITC = 0.122), and change of residence (CITC = 0.045)] (Table 3).

**Table 3:** Cronbach’s alpha values for STOP-SRiFs and STOP-SReFS scales

	Adolescent sample	Parent sample	Clinician sample
*Cronbach’s alpha*			
STOP-SRiFS	0.864	0.842	0.722
STOP-SReFS	0.775	0.808	0.808
2. *Test*–*retest reliability* The results showed that there was good temporal stability (test–retest reliability), through the intra-class correlation coefficients between the STOP-SRiFS sub-scales scores at the first and second administration (within 3 weeks ~ 19 Days). All the intra-class correlations were good (> 0.600) (see Table 4).

**Table 4:** Intraclass correlation coefficients from the STOP-SRiFS and STOP-SReFS

	Adolescent	Parent	Clinician
*Intraclass correlation coefficient (STOP-SRiFS)*			
Anxiety and depression risk	0.879	0.933	0.884
Substance misuse risk	0.932	0.980	0.901
Interpersonal risk	0.733	0.606	0.961
Chronic risk	0.736	0.611	0.842
Risk due to life events	0.877	0.909	0.943
*Intraclass correlation coefficient (STOP-SReFS)*			
Cognitive resilience	0.846	0.547	0.866
Interpersonal resilience	0.814	0.764	0.882
3. *Inter*-*rater reliability* Table 5 presents the inter-version correlations between the different STOP-SRiFS sub-scales for the adolescent, parent, and clinician versions, and shows that they were good (Pearson’s correlation coefficient threshold was set at *r *> 0.200 [27]).

**Table 5:** Inter-version correlation coefficients from the STOP-SReFS and the STOP-SRiFS sub-scales

	Adolescent	Parent	Clinician
*Inter-version correlations for STOP-SRiFS*			
Anxiety and depression risk sub-scale			
Adolescent	1	0.537**	0.643**
Parent	0.537**	1	0.444**
Clinician	0.643**	0.444**	1
Substance misuse risk sub-scale			
Adolescent	1	0.813**	0.837**
Parent	0.813**	1	0.781**
Clinician	0.837**	0.781**	1
Interpersonal risk sub-scale			
Adolescent	1	0.548**	0.649**
Parent	0.548**	1	0.490**
Clinician	0.649**	0.490**	1
Chronic risk sub-scale			
Adolescent	1	0.314**	0.469**
Parent	0.314**	1	0.090
Clinician	0.469**	0.090	1
Risk due to life events sub-scale			
Adolescent	1	0.197*	0.136
Parent	0.197*	1	0.044
Clinician	0.136	0.044	1
*Inter-version correlations for STOP-SReFS*			
Cognitive resilience sub-scale			
Adolescent	1	0.105	0.623**
Parent	0.105	1	− 0.035
Clinician	0.623**	− 0.035	1
Interpersonal resilience sub-scale			
Adolescent	1	− 0.116	0.574**
Parent	− 0.116	1	0.159
Clinician	0.574**	0.159	1

*Correlation is significant at the 0.05 level (two-tailed)

**Correlation is significant at the 0.01 level (two-tailed)
4. *Exploratory factor analysis* The items that showed poor-corrected item-total correlation (CITC) for the Parent and Clinician versions of the scale were also excluded by the EFA and, therefore, from any subsequent psychometric analysis. The experts in childhood suicidality who reviewed these results recommended that the aforementioned items, given their clinical relevance, should continue to be administered at the end of the scale as extra items, which will not be used in the scoring of the scales. As shown in Table 6, EFA for the Adolescent version of the STOP-SRiFS (consisting 21 risk factor domains), a 5-factor model was determined to best fit the data based on the screen plot. The Kaiser–Meyer–Olikin (KMO) was 0.816 (*X*^2^ = 1787.257; Bartlett’s test of sphericity *p* ≤ 0.001; *df* = 210). The Parent version of the scale without the two items excluded based on the Corrected Item-Total Correlation showed again that the best model to explain the structure of the scale is again a 5-factor model. The KMO was 0.720 (*X*^2^ = 727.698; Bartlett’s test of sphericity *p* ≤ 0.001, *df* = 171). The results of the EFA for the parent version of the STOP-SRiFS showed that the item about the ‘misuse of other drugs’ had the highest loading on the factor which assessed the sub-domains concerning risk due to life events (0.216). The second highest loading for this item (0.179) was on the factor which assessed the sub-domains concerning substance misuse risk. Based on the clinical judgment of experts in child and adolescent mental health, it was decided that it was clinically relevant for this item to be part of the factor about substance misuse risk (see Table 6 for more details about the STOP-SRiFS factor structure). The Clinician version of the scale (5 factors) also presented with a KMO of 0.772 (*X*^2^ = 895.861; Bartlett’s test of sphericity *p* ≤ 0.001, *df* = 136). Based on the pattern of risk factors domain loading, the five factors were named as: (1) anxiety and depression risk, (2) substance misuse risk, (3) interpersonal risk, (4) chronic risk, and (5) risk due to life events. These factors capture the clinical risk clusters in adolescents with suicidal ideations or behaviours.

**Table 6:** Exploratory factor analysis for the adolescent, parent, and clinician version of the STOP-SRiFS and STOP-SReFS

STOP-SRiFS	Anxiety and depression risk	Substance misuse risk	Interpersonal risk	Chronic risk	Risk due to life events
*Adolescent*					
Depressive thinking	**0.960**	− 0.076	− 0.011	0.001	− 0.057
Pessimism	**0.928**	0.007	0.028	− 0.091	− 0.003
Low self-esteem	**0.905**	0.009	0.017	− 0.126	0.080
Depressive mood	**0.850**	0.027	0.020	0.027	− 0.090
Anxiety	**0.780**	0.036	− 0.075	0.139	− 0.015
Worrying about school performance	**0.472**	0.036	0.127	0.026	0.091
Misuse of Cannabis	0.000	**0.881**	− 0.027	− 0.039	− 0.087
Smoking	0.073	**0.810**	− 0.096	0.066	0.019
Exaggerated use of alcohol	− 0.002	**0.598**	0.076	− 0.170	0.092
Misuse of other drugs	− 0.025	**0.396**	0.019	− 0.028	0.088
Relationship breakup	− 0.090	**0.345**	**0.318**	0.187	− 0.132
Searching Internet sites about suicide	0.140	− 0.035	**0.815**	− 0.154	− 0.056
History of suicidal attempt	− 0.032	0.024	**0.713**	0.131	− 0.026
Bullying	0.133	− 0.115	**0.247**	0.137	0.128
Discomfort with sexual identity	− 0.012	0.093	**0.231**	− 0.093	0.221
Chronic physical condition that produces discomfort	0.006	− 0.100	− 0.123	**0.581**	− 0.038
Presence of chronic pain	0.079	− 0.097	0.135	**0.527**	− 0.019
Preoccupation about death of close one	− 0.135	0.091	0.049	**0.368**	0.126
Presence of problems at home	0.281	0.103	0.014	**0.301**	0.197
Change of residence	− 0.065	− 0.011	0.040	− 0.010	**0.759**
History of completed suicide in the family	0.099	0.061	− 0.151	0.088	**0.350**
*Parent*					
Pessimism	**0.923**	− 0.142	0.051	− 0.022	− 0.106
Depressive mood	**0.863**	0.039	0.002	0.004	− 0.027
Low self-esteem	**0.847**	0.013	0.058	− 0.034	0.065
Anxiety	**0.830**	0.052	− 0.016	− 0.017	− 0.015
Depressive thinking	**0.677**	− 0.051	0.057	0.157	0.227
Worrying about school performance	**0.266**	0.043	0.219	0.073	0.061
Misuse of cannabis	0.043	**0.911**	− 0.069	− 0.151	0.031
Smoking	0.071	**0.638**	0.058	0.122	0.033
Exaggerated use of alcohol	− 0.278	**0.411**	0.221	0.169	− 0.024
Presence of chronic pain	0.101	− 0.003	**0.708**	0.110	− 0.207
Chronic physical condition that produces discomfort	0.089	0.081	**0.588**	− 0.223	− 0.110
Searching Internet sites about suicide	0.039	− 0.015	0.007	**0.795**	− 0.076
History of suicidal attempt	0.211	0.190	0.015	**0.430**	0.013
Presence of problems at home	− 0.012	0.000	0.210	− 0.266	**0.735**
Relationship breakup	− 0.066	− 0.027	0.322	0.175	**0.399**
Preoccupation about death of close one	− 0.067	− 0.054	− 0.175	0.268	**0.393**
History of completed suicide in the family	0.131	0.038	− 0.235	− 0.088	**0.327**
Bullying	0.226	0.001	− 0.153	0.183	**0.230**
Misuse of other drugs	0.038	**0.179**	− 0.145	0.056	0.216
*Clinician*					
Depressive Thinking	**0.896**	− 0.071	0.128	− 0.104	− 0.047
Pessimism	**0.830**	0.079	− 0.074	− 0.017	− 0.072
Low self-esteem	**0.759**	0.053	− 0.183	− 0.013	0.038
Depressive mood	**0.754**	0.055	0.057	0.064	− 0.136
Anxiety	**0.521**	− 0.126	0.009	0.037	0.358
Misuse of cannabis	0.031	**0.823**	− 0.050	0.012	0.025
Smoking	0.072	**0.780**	− 0.120	− 0.029	0.118
Exaggerated use of alcohol	− 0.035	**0.662**	0.146	− 0.062	0.025
History of suicidal attempt	− 0.019	− 0.027	**0.578**	− 0.055	0.052
Discomfort with sexual identity	− 0.083	− 0.011	**0.392**	− 0.078	0.080
Searching Internet sites about suicide	0.209	0.063	**0.286**	0.202	− 0.032
Presence of problems at home	0.065	− 0.028	− 0.149	**0.521**	− 0.065
Presence of chronic pain	− 0.093	− 0.064	0.012	**0.346**	0.004
Relationship breakup	− 0.056	0.231	0.158	**0.309**	− 0.016
History of completed suicide in the family	0.021	0.160	0.121	− 0.187	**0.357**
Preoccupation about death of close one	− 0.194	0.119	− 0.001	0.123	**0.341**
Worrying about school performance	0.175	− 0.068	0.048	0.173	**0.289**

STOP-SReFS	Interpersonal resilience	Cognitive resilience
*Adolescent*		
Friendships	**0.787**	− 0.135
Optimism	**0.672**	0.070
Hobbies	**0.606**	− 0.022
Internal control	**0.476**	0.017
Empathy	**0.407**	0.271
Religious beliefs	**0.234**	0.075
Family connectiveness	− 0.144	**0.920**
Social environment	0.373	**0.488**
External control	0.028	**0.217**
*Parent*		
Social environment	**1.063**	− 0.133
Family connectiveness	**0.505**	0.272
Internal control	**0.335**	0.119
Empathy	− 0.030	**0.788**
Optimism	0.080	**0.634**
Friendships	0.254	**0.438**
Religious beliefs	− 0.022	**0.334**
External control	0.083	**0.331**
Hobbies	0.275	**0.321**
*Clinician*		
Social environment	− 0.146	**0.992**
Family connectiveness	− 0.087	**0.743**
Friendships	0.196	**0.509**
Hobbies	0.191	**0.487**
Empathy	0.281	**0.460**
External control	0.049	**0.335**
Optimism	**0.808**	0.010
Religious beliefs	**0.445**	0.021
Internal control	**0.423**	− 0.030

Extraction method: principal axis factoring, and promax rotation

Numbers in bold indicate the item that the factor belongs to
5. *Content validity* As predicted, the correlations between the sub-scales of STOP-SRiFS adolescent version and the C-SSRS total score were significant, indicating that increased risk was associated with increased C-SSRS total score (Table 7). Broadly speaking, the STOP-SRiFS sub-scale scores in the parent and clinician versions were similarly correlated with the C-SSRS total score. The sub-scale scores that did not reach significance were those which would be rated differently by the parents and clinicians in comparison to the adolescent (Table 7).

**Table 7:** Correlations between STOP-SReFS and STOP-SRiFS sub-scales, and the C-SSRS total score

	Adolescent	Parent	Clinician
*Columbia-suicide severity rating scale correlations*			
STOP-cognitive resilience sub-scale	− 0.275**	− 0.070	− 0.143*
STOP-interpersonal resilience sub-scale	− 0.117	− 0.132	− 0.046
STOP-anxiety and depression risk sub-scale	0.610**	0.426**	0.497**
STOP-substance misuse risk sub-scale	0.221**	0.103	0.097
STOP-interpersonal risk sub-scale	0.491**	0.450**	0.472**
STOP-chronic risk sub-scale	0.287**	− 0.029	0.153*
STOP-risk due to life events sub-scale	0.088	0.193**	0.178**

**Correlation is significant at the 0.01 level (two-tailed)

*Correlation is significant at the 0.05 level (two-tailed)

### STOP-SReFS

1. *Construct validity* The Cronbach’s alpha values for all the versions of the STOP-SReFS (Adolescent: 0.775; Parent: 0.808; Clinician: 0.808) (Table 3) indicate good internal consistency of the scale (Table 3).
2. *Test*–*retest reliability* The results showed a good temporal stability (test–retest reliability). This was assessed in Sample 1 through the intra-class correlation coefficients between the STOP-SReFS sub-scales scores at the first and second administration (within 3 weeks ~ 19 days). These results showed that all intra-class correlations were above the acceptance threshold (> 0.600), except for the parent cognitive resilience scale which was below the threshold (0.547) (see Table 4). This is understandable, because suicidality risk factors can change even in the short period used for the test–retest.
3. *Inter*-*rater reliability* Table 5 presents the inter-version correlations between the different STOP-SRiFS and STOP-SReFS sub-scales for the Adolescent, Parent, and Clinician versions, which were all acceptable.
4. *Exploratory factor analysis* As shown in Table 6, EFA for the Adolescent version of the STOP-SReFS identified that a two-factor model was the best fit (the KMO was 0.769 (*X*^2^ = 511.748; Bartlett’s test of sphericity *p* ≤ 0.001, *df* = 36). The EFA of the STOP-SReFS Parent version also showed that the best model to explain the structure of the scale was a two-factor model (the KMO was 0.819 (*X*^2^ = 446.362; Bartlett’s test of sphericity *p* ≤ 0.001, *df* = 36). The EFA of the STOP-SReFS Clinician version was similar and had a KMO of 0.813 (*X*^2^ = 572.156; Bartlett’s test of sphericity *p* ≤ 0.001, *df* = 36). Based on the pattern of resilience factors domain loading, the two factors were named: (1) interpersonal resilience and (2) cognitive resilience. These resilience factors are in keeping with known protective factors. The EFA revealed that the scales were not unidimensional and, therefore, precludes the use of a total score. In view of this, correlations between the STOP-SReFS sub-scales and their correlations with the C-SSRS total score were performed.
5. *Content validity* Correlations between the STOP-SRiFS and STOP-SReFS sub-scales, and the C-SSRS total score are presented in Table 7. As expected, the C-SSRS negatively correlated with the STOP-SReFS (captures protective factors) cognitive resilience sub-scale for the adolescent (*r* = − 0.275). However, the clinician (*r* = − 0.143) versions of the scale did not meet the threshold of *r* > 0.200 [26] (Table 7). The STOP-SReFS Interpersonal resilience sub-scale correlations were all negative, but none of them were significantly different to the C-SSRS total scores for either the adolescent, parent, or clinician versions of the scales.

## Discussion

Despite progress made in suicidality research, the risk and resilience factors involved in suicidal behaviour and ideation remain poorly understood. The present study describes the development and the subsequent psychometric validation of two scales: the STOP-Suicidality Risk Factors Scale (STOP-SRiFS) and the STOP-Suicidality Resilience Factors Scale (STOP-SReFS)—two web-based instruments that measure elements of suicidality on the web-based HealthTracker™ system. The measurement properties of the two instruments were assessed using the consensus-based standards for the selection of health status Measurement instruments (COSMIN) [28]. The COSMIN checklist was used to structure the layout of the manuscript when reporting a study describing psychometric instruments. Using this approach, the psychometric analyses revealed that the STOP-SReFS and the STOP-SRiFS were reliable and valid instruments for assessing suicidality risk and resilience factors in adolescents. The fact that the STOP-SRiFS and the STOP-SReFS are more age-specific scales, which have been designed and worded specifically for the adolescent population, and that they can be completed online, decreasing completion time and ensuring accessibility at all times, increases their potential applicability in an adolescent population [29]. As suicidal behaviour depends on diverse clinical, psychological, sociological, and biological factors, the consensus is that a multi-informant evaluation is strongly recommended [21]. Furthermore, adolescents who are a particularly high-risk group for suicidality differ from the adult population and need a deeper, wider, and multi-dimensional approach [30]. The study of cross-informant agreement has been shown to be useful in obtaining a more detailed understanding of the adolescent population [31] as they usually tend to not report the same information as their parents, teachers, or clinicians. In this study, results in adolescents and parents showed a good correlation, contrary to some studies that report low agreement between parents and adolescents [32].

The threshold for the minimum loading for EFA was set at > 0.200. Thresholds in the region of > 0.200 have been cited in the literature [33, 34] and we have previously used a similar threshold for factor loading to validate and assess the psychometric properties of a parent version of a neuropsychiatric scale [35]. In the context of the present study, we set a threshold of > 0.200 so that the factor loading would best reflect the phenomenon of interest in accordance with our sample size, clinical judgement, and exploratory nature of the study.

Suicide risk factors have been widely studied, whilst the study of protective factors has been usually neglected. However, in the past years, there has been an increasing interest in incorporating the concept of resilience into the suicidality paradigm [36]. The identification of specific risk and resilience factors in young people could help to develop personalized therapeutic strategies, in which treatment is tailored to the personal needs of each patient. In addition, this could lead to the development of targeted interventions for some of these risk and/or resilience factors, for example, intervention programs aimed at improving the family connectedness. This knowledge may lead to actions and changes which can have an impact on the suicide rates as shown in the Youth Aware of Mental Health Programme (YAM), a manualized, universal school-based intervention which has shown efficacy in reducing the number of suicide attempts and severe suicidal ideation in adolescents [16]. The SEYLE trial, which has been recruiting a large number of European adolescents, has also addressed these issues, concluding that screening is an efficient method to refer subjects in need of treatment [16].

The Internet has become a public and accessible information exchange forum for individuals. The use of new technologies could innovate healthcare, i.e., a web-based version of a questionnaire may enhance perceptions of privacy and confidentiality, which may improve honesty of responses, particularly when less socially desirable, especially to those items related to emotions [37].

As far as we are aware of, this is the first attempt to assess risk and resilience factors related to suicidality in the adolescent population using web-based measures, and accounting for different sources of information. The thorough methodology employed, the sample size, the focus groups in which all interested parties were involved in co-designing the scales, the external scientific supervision by experts in the field, and its applicability to multiple pathologies and settings offered added value to this study.

## Limitations

There are limitations to this study that need to be considered. To identify subjects at risk, a positive and undefined answer to the screening questionnaire of the STOP-Suicidality Assessment Scale (STOP-SAS) [21] was necessary for patients to be allocated the full STOP-SRiFS and STOP-SReFS. Since the aim of the study was to develop a universal instrument, we did not account for the effect of diagnosis and sex on these risk and protective factors. Moreover, not being able to substitute the missing values in the database with estimations led to a reduced sample size.

## Conclusion

The current study suggests that the STOP-SRiFS and the STOP-SReFS scales are viable instruments to assess risk and resilience factors in young people. They can be used to identify subgroups in the adolescent population who may need targeted intervention. In this vein, the STOP-SRiFS and the STOP-SReFS could be used as effective risk stratification tools to provide a multi-informant view on adolescent risk for suicidality and maybe of value for the assessment of suicidality in clinical trials. This sentiment has been echoed by others, who have highlighted the need for improving the detection and assessment of suicidality in clinical trials [38]. Moreover, the identification of subpopulations with a personalized level of specific risk and protective factors could guide personalized interventions, which ultimately may help to reduce suicide rates and improve prognosis in paediatric populations.
